# Effective Local Methotrexate Therapy for Interstitial Ectopic Pregnancy: A Case Report and Review of the Literature

**DOI:** 10.1155/crog/5557768

**Published:** 2026-01-28

**Authors:** Nasser Saleh Alabbad, Mohammed Jassim Alhassan, Jawad S. Alnajjar, Khadijah J. Alhassan, Azhar Abdulmohsen Al Dehneen

**Affiliations:** ^1^ Maternal Fetal Consultant, King Faisal General Hospital, Alahsa, Saudi Arabia; ^2^ College of Medicine, King Faisal University, Al Ahsa, Saudi Arabia, kfu.edu.sa; ^3^ Senior Registrar of Obstetrics and Gynecology, Maternity And Children Hospital, Al Ahsa, Saudi Arabia, moh.gov.sa; ^4^ Consultant of Obstetrics and Gynecology, King Faisal General Hospital, Al Ahsa, Saudi Arabia

**Keywords:** ectopic pregnancy, extrauterine pregnancy, gynecological emergencies, high-risk pregnancy, interstitial pregnancy

## Abstract

**Introduction:**

Interstitial ectopic pregnancy is a rare and potentially life‐threatening condition that occurs when a fertilized ovum implants in the interstitial (intramural) portion of the fallopian tube within the myometrium. It accounts for 2%–4% of all ectopic pregnancies and poses significant risks due to the potential for rupture and severe hemorrhage.

**Case Presentation:**

We report the case of a 34‐year‐old woman, gravida 5 para 3 + 1, living 4, who presented with right iliac fossa pain at 5 weeks of gestation. Ultrasonography and elevated *β*‐hCG levels revealed a suspicious interstitial ectopic pregnancy. Initial treatment with systemic methotrexate was administered; however, due to plateauing *β*‐hCG levels, a second dose of methotrexate was given via direct injection into the ectopic site. Follow‐up monitoring demonstrated a significant reduction in *β*‐hCG levels, resulting in the successful resolution of the ectopic pregnancy.

**Conclusion:**

This case highlights the successful treatment of interstitial ectopic pregnancy with local methotrexate injection, emphasizing the importance of early diagnosis and timely intervention. Future studies with larger sample sizes and standardized treatment criteria are recommended.

## 1. Introduction

Ectopic pregnancy refers to the implantation of a fertilized ovum outside the endometrial cavity [1]. Rupture of an ectopic pregnancy remains a major cause of first‐trimester maternal mortality, accounting for up to 10% of all pregnancy‐related deaths [1]. Most ectopic pregnancies occur within the fallopian tube; however, implantation may also take place in interstitial, cornual, ovarian, cervical, or abdominal sites [2]. Rarely, ectopic pregnancies have been reported in highly unusual locations such as the liver, pelvic wall, and retroperitoneum [3, 4]. Interstitial ectopic pregnancy is diagnosed on transvaginal ultrasound by an eccentrically located gestational sac in the uterine fundus separated from the endometrial cavity (empty cavity), a thin myometrial mantle typically < 5 mm around the sac and/or the sac positioned > 1 cm lateral to the most lateral edge of the uterine cavity, and the pathognomonic “interstitial line sign”—an echogenic line extending from the endometrium to the sac representing the interstitial portion of the tube [5, 6].

Differential diagnosis includes angular pregnancy (implantation medial to the uterotubal junction inside the endometrial cavity with lateral cavity enlargement rather than an outside‐myometrium location), intramural pregnancy (very rare implantation within the myometrium without a clear tubal tract), and subserosal or myoma‐related implantation (implantation on the serosal surface or within a fibroid)—distinctions that rely on demonstrating whether the sac communicates with or displaces the endometrial cavity, the thickness/location of intervening myometrium, and the presence/absence of the interstitial line; when 2‐D sonography is equivocal, 3‐D ultrasound or MRI improves localization [7–9]. Management strategies depend largely on the patient’s clinical stability and the presence or absence of rupture [10]. Here, we describe a rare case of an unruptured interstitial ectopic pregnancy successfully treated with a local methotrexate (MTX) injection directly into the ectopic sac.

## 2. Case Presentation

A 34‐year‐old White female, G5 P3 + 1 L4, presented with a history of three previous cesarean sections and one first‐trimester abortion. Her past medical history was unremarkable, but her surgical history included hemorrhoidectomy and three cesarean deliveries. Her first cesarean was performed at term due to cord presentation, the second pregnancy ended in a first‐trimester abortion, the third was a cesarean at 34 weeks due to scar tenderness in a twin pregnancy, and the fourth was a cesarean at term 3 years ago. She is currently in her fifth pregnancy, which occurred spontaneously.

**Figure 1:**
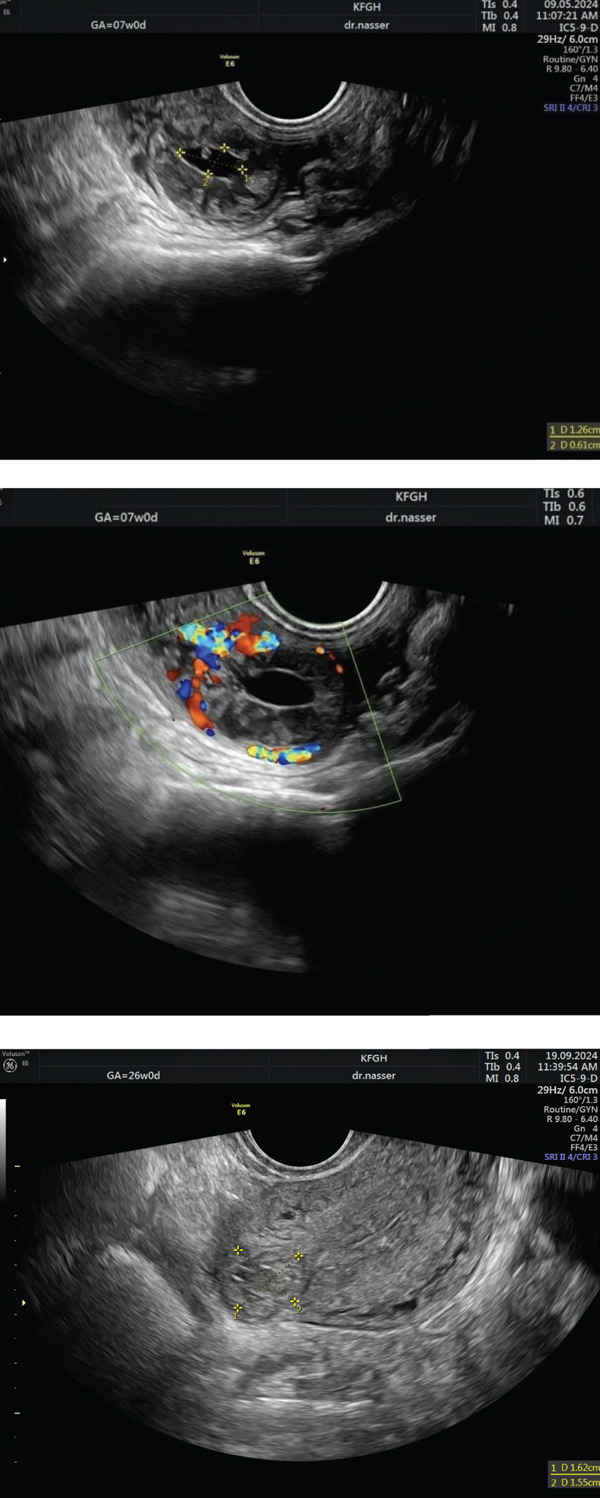
Ultrasound image of an interstitial ectopic pregnancy before the initiation of methotrexate therapy.

At 5 weeks gestational age (GA), the patient began experiencing throbbing pain in the right iliac fossa, unaccompanied by nausea, vomiting, shoulder pain, dizziness, or vaginal spotting. By 6 weeks GA, the pain persisted, prompting her to visit the emergency room for further evaluation. Physical examination revealed tenderness in the right iliac fossa. A pelvic examination showed a closed cervix with cervical excitation. Figure 1 illustrates an ultrasound showing a normal‐sized anteverted uterus with a central thin fluid in the uterine cavity and an 11‐mm thickened endometrial lining. The right adnexa displayed a 20 × 13 mm ring‐like structure with increased vascularity, suggestive of an ectopic pregnancy, while the left adnexa and the pouch of Douglas were unremarkable. Initial serum *β*‐hCG was 3703 mIU/mL.

**Figure 2:**
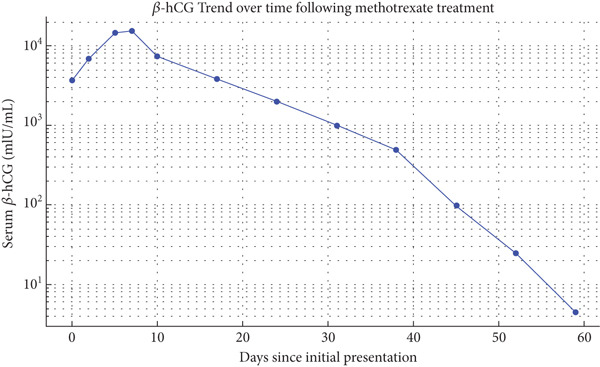
Serial *β*‐hCG levels following methotrexate therapy for interstitial ectopic pregnancy, showing initial plateau and subsequent progressive decline to resolution within 7 weeks.

**Figure 3:**
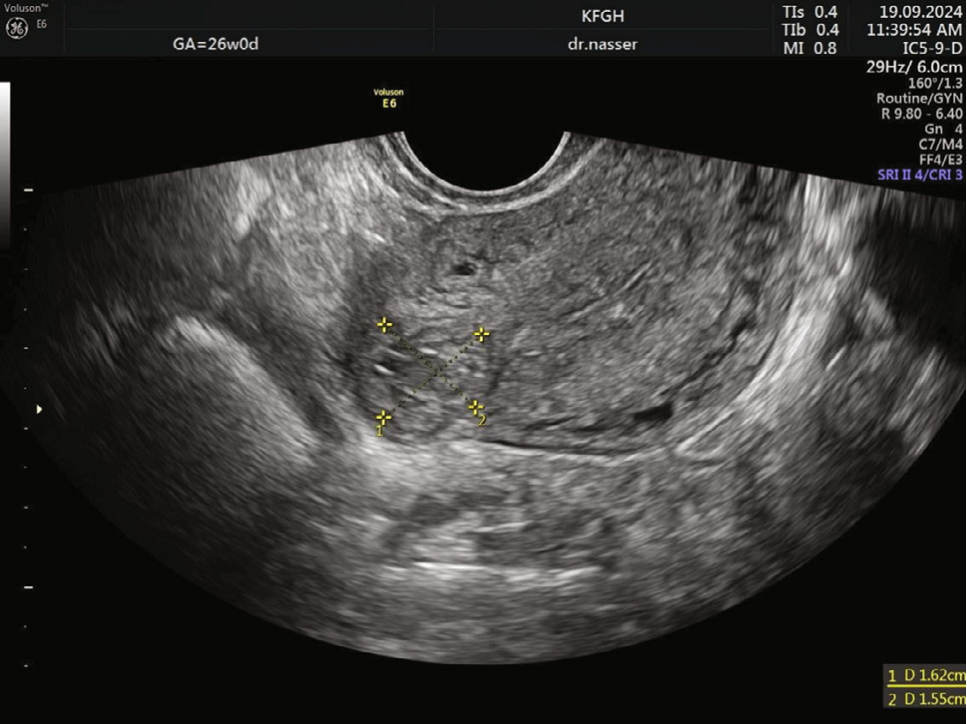
Ultrasound image of an interstitial ectopic pregnancy 3 months after the initiation of methotrexate therapy.

Two days later, a repeat *β*‐hCG level was 7000 mIU/mL, and a follow‐up US showed unchanged findings. The patient was diagnosed with an interstitial ectopic pregnancy. The patient was counseled regarding available management options; she preferred a non‐surgical and less invasive approach, so she received a single dose of intramuscular 83 mg of MTX, but follow‐up *β*‐hCG levels on Day 3 (14,705 mIU/mL) and Day 7 (15,650 mIU/mL) showed plateauing between Days 3 and 7. Due to insufficient response, a second dose of 83 mg MTX was administered directly to the ectopic pregnancy trans‐vaginally. Subsequent *β*‐hCG levels on Day 3 (7430 mIU/mL) and Day 7 (3873 mIU/mL) showed a significant drop of more than 15%. The patient continued weekly *β*‐hCG monitoring, which showed a steady decline to below 5 mIU/mL within 7 weeks, confirming complete resolution of the interstitial ectopic pregnancy. Figure 2 depicts this trend, showing an initial plateau after the first MTX dose, followed by a consistent decline after the second, indicating a favorable response to treatment. Finally, 3 months after therapy initiation, a follow‐up transvaginal ultrasound (Figure 3) confirmed complete resolution of the interstitial ectopic pregnancy, with no side effects, complications, or need for blood transfusion reported throughout the course of management.

## 3. Discussion

Interstitial pregnancy arises from implantation of the blastocyst in the intramural portion of the fallopian tube within the myometrium [11, 12]. It accounts for 2%–4% of all ectopic pregnancies and carries a high risk of catastrophic hemorrhage because of its rich vascular supply [13]. Clinically, interstitial pregnancy typically presents with vaginal bleeding or abdominal pain during the first trimester, although it may remain asymptomatic in the early stages [12]. The interstitial portion of the fallopian tube is approximately 0.7 mm in width and 1–2 cm in length. The surrounding myometrium allows the pregnancy to progress further than other ectopic sites, often into the second trimester, but rupture at this advanced stage can result in massive hemorrhage with a reported mortality rate of up to 2% [14]. The terminology of *interstitial* and *cornual* pregnancy is often used interchangeably, though the distinction is clinically important. Interstitial pregnancy occurs in the intramural segment of a normal uterus, whereas cornual pregnancy refers to implantation in a rudimentary uterine horn or malformed uterus [14].

Diagnosis relies on careful imaging and a high index of clinical suspicion. Ultrasonography, serial serum *β*‐hCG monitoring, and heightened awareness have improved the success rates of conservative management [13]. Sonographically, a rim of myometrial tissue surrounding an eccentrically located gestational sac, with an echogenic line extending from the sac to the endometrial cavity, is highly specific for interstitial pregnancy [11]. However, differentiation from an early intrauterine gestation can be challenging, particularly in early scans [11] [15],

.Early recognition and consistent terminology are essential for appropriate management and patient counseling. Risk factors for interstitial pregnancy include tubal anomalies such as endometriosis or fibroids, previous pelvic inflammatory disease, prior ectopic pregnancy, pelvic or uterine surgery (including previous cesarean delivery), and the use of assisted reproductive technologies [12]. Awareness of these factors facilitates early detection and helps prevent recurrence. Because symptoms are often nonspecific, interstitial ectopic pregnancy remains a diagnostic and therapeutic challenge in modern gynecology [16].

Conservative medical treatment is increasingly preferred for selected stable patients. Appropriate criteria for medical therapy include early gestation, an ectopic mass<4 cm in diameter, *β* − hCG < 10,000 IU/L, and absence of fetal cardiac activity or anatomical risk factors [10]. Despite several therapeutic advances, evidence supporting the use of local MTX in these atypical sites remains limited [17, 18]. In a 2017 case series, MTX was not considered the first‐line treatment in two patients—one with very high *β*‐hCG levels and another with a viable embryo showing cardiac activity—both representing relative contraindications to medical therapy [10]. When anatomical abnormalities are present or recurrence occurs, surgical management such as cornual wedge resection or cornuotomy is generally preferred [19]. Other procedures, including salpingectomy, endoloop ligation, or laparoscopic resection and curettage, may also be used but remain limited by scarce evidence [19].

MTX remains the primary medical treatment for interstitial ectopic pregnancy due to its ability to target rapidly dividing trophoblastic tissue, offering a fertility‐preserving alternative to surgery. Combination regimens, such as MTX with mifepristone, have been explored as conservative options to potentially increase treatment efficacy. Mifepristone, a progesterone receptor antagonist, enhances the effect of MTX by promoting decidual breakdown and increasing trophoblast sensitivity to cytotoxic therapy. In a recent review, Dealberti et al. reported that most interstitial pregnancies treated with this combination resolved successfully; however, complications occurred in a minority of cases. These included hemoperitoneum requiring surgical intervention in two patients and a rare uterine arteriovenous malformation managed with embolization. Both drugs carry potential adverse effects. MTX may cause gastrointestinal symptoms, transient liver toxicity, myelosuppression, and, rarely, pulmonary complications. Mifepristone can lead to prolonged or heavy vaginal bleeding, uterine cramping, and hypersensitivity reactions. When combined, careful monitoring is essential to detect both treatment‐ and drug‐related complications. These findings highlight that MTX‐based combination therapy, while fertility‐preserving and effective, requires careful patient selection and close follow‐up to manage the risk of significant bleeding and other adverse outcomes [20].

Our patient met accepted criteria for local MTX injection, including informed consent, *β*‐hCG < 15,000 mIU/mL, absence of fetal cardiac activity, sac size < 4 cm, no intra‐abdominal bleeding, and availability of an experienced operator with proper equipment. Successful outcomes depend not only on these factors but also on strict adherence to follow‐up and monitoring. Evidence supporting the effectiveness of local MTX therapy remains limited. A report involving 10 cases of interstitial pregnancy compared systemic and local MTX administration; the local approach achieved a 100% success rate, while the systemic approach had an 80% success rate [17]. Furthermore, in selected cases with detectable fetal cardiac activity, intracardiac potassium chloride injection before MTX administration has been described with favorable results [21]. However, further studies are needed to validate selection criteria and confirm the efficacy of local MTX therapy in interstitial ectopic pregnancy.

## 4. Conclusion

Early diagnosis and targeted MTX therapy can successfully treat interstitial ectopic pregnancy in stable patients. Local MTX injection offers a safe, minimally invasive, and fertility‐preserving alternative to surgery when applied with strict monitoring.

## Ethics Statement

Ethical approval was given by the Maternity and Children Hospital.

## Consent

Written informed consent was obtained from the patient for the publication of this case report.

## Disclosure

All authors approved it for publication.

## Conflicts of Interest

The authors declare no conflicts of interest.

## Author Contributions

All authors listed have made a direct and intellectual contribution to the work.

## Funding

No funding was received for this manuscript.

## Data Availability

The data that support the findings of this study are available on request from the corresponding author. The data are not publicly available due to privacy or ethical restrictions. The data supporting the conclusions of this case report are derived from the clinical records of the patient involved. Due to the personal and sensitive nature of these medical records, and to protect patient confidentiality, the data are not publicly available. Access to the data may be granted by the corresponding author upon reasonable request, subject to institutional and ethical approval.
